# Medical Items

**Published:** 1898-05

**Authors:** 


### MEDICAL ITEMS.

---

Dr. James H. Bryant, of Talbotton, Ga., died April 14.

---

Married.—Dr. O. N. Pendergrass and Miss Metie Connor, of Social Circle, April 28.

---

Colleges.—Our readers should notice the college advertisements in this and succeeding issues in order to know where to send their students.

---

There are three men in our social system who cannot respect or value the world—the physician, the lawyer, and the priest. They wear black; perhaps in mourning for all virtues and all illusions.—*Balzac.*

---

Dr. C. S. Harris, of Rome, died April 18, in his seventy-second year. He had been a resident of Rome for forty years, and was a respected and successful practitioner. During the civil war he was a surgeon in Hood's army.

---

The *International Magazine* is responsible for the statement that of 217,000 prescriptions written in Chicago, New York, Boston, Washington, Baltimore, Denver, San Francisco, New Orleans, and St. Louis, 11.25 per cent. called for proprietary articles.—*Ex.*

---

Dr. Chas. Brigham, of San Francisco, has lately successfully performed esophago-enterostomy, after Schlatter's method. The patient was a woman, sixty-six years of age, the date of the operation being about two months ago. She has made a good recovery, and her digestive functions do not appear at all disturbed.

ARTHUR ORTON, better known to fame as the false "Sir Roger Tichborne," has just died in London. It will be remembered that English courts were occupied five years with this very remarkable case of confused identity. Finally the claimant was exposed and condemned to fourteen years of penal servitude.

---

DR. GEO. HENRY FOX, Professor of Dermatology in the College of Physicians and Surgeons, New York, gave the Atlanta Society of Medicine a talk upon syphilitic eruptions at the meeting of April 7. His remarks were illustrated with stereopticon views of the various syphilitic manifestations. The doctor's comments were eminently instructive and the pictures were excellent.

---

FOR the Denver meeting of the American Medical Association the Western Passenger Association has granted a rate to Denver and return of one-half fare, plus $2.00, thirty-day limit, for business from Chicago, St. Louis and intermediate points. Tickets on sale June 2d, 4th and 5th east of the Missouri River; 5th and 6th west of the Missouri River. A round trip rate of $20.00, thirty-day limit, from Ogden and Salt Lake, is also announced. Application for similar rates has been made to all other passenger associations and to railroads not controlled by them.

---

THE regular Board of Medical Examiners met in annual session in Atlanta, March 31st. Sixty-four applicants were examined for license. Four failed—two from the Atlanta Medical College, one from the Southern Medical College, and one from the Tennessee Medical College. Dr. J. B. S. Holmes, of Atlanta, was elected President, and Dr. E. R. Anthony, of Griffin, Secretary and Treasurer for the ensuing year.

The board also met in Augusta April 2. Twenty-nine applicants were examined. Two failed—one from the Medical College of Georgia (Augusta), and one from Meharry Medical College (Nashville).

IT will be remembered that when young Gammon was killed in a football game in Atlanta last fall and bills were afterwards introduced in the Legislature prohibiting further match-games in the State, the young man's mother, in a public letter, requested that "the death of her son should not serve as an argument against the development of an athletic education in the university." This letter, it is said, has been widely published in this country, and the *Progrès Medical*, of Paris, says that it has been translated in the press of continental Europe. The last named journal says: "That's the kind of mothers they have in America. Such mothers are the makers of true men."

---

THE medical excursion in June will leave Denver for Salt Lake City—the Zion of the new world—on the last day of the meeting, and the two successive days via the Rio Grande Western Railway in connection with the D. & R. G. and Colorado Midland lines. The rate will be but $18.00 for the round trip, offering a trip of 1,500 miles through the Rocky and Wasatch Mountains. No European trip of equal length can compare with it in grandeur or wealth of novel interest. Salt Lake City and vicinity are one grand sanitarium. The Great Salt Lake or Dead Sea of America, with its magnificent bathing resort, the hot and warm springs, drives, parks, canyons and reserves are all located in or about the city. Send two cents to F. A. Wadleigh, Salt Lake City, for copy of pamphlet.

---

THE nineteenth annual report of the Atlanta Board of Health (for 1897) is before us. It shows, among other things, an annual death-rate of 18.26 per thousand, the population consisting of 60,000 whites and 40,000 colored. There were 806 deaths among the whites and 1,020 among the colored. Annual death-rate for the whites, 13.43; for the blacks, 25.50 per thousand. There were 214 deaths from consumption, or 11.71 per cent. of the total number of deaths. There were 54 deaths from typhoid fever, confined practically to those persons who habitually drink well-water. Within the year there were 247 cases of smallpox, with three deaths. Of the 214 deaths from tuberculosis, 66 were white and 148 colored.

The report displays a lack of proof-reading skill which is anything but creditable to both compiler and printer. Under "Causes of Death" we learn, for instance, that one person died of "fragilitis ossimo"; another had "multiper scolisis"; another, "maltruition"; one died from enlarged "protestate"; another from "intussus caption," and another from rupture of the "Gaul Bladder." "Puturis" and "inflammation of the setamus," whatever those diseases may be, also destroyed a victim each.

---

The third annual meeting of the Western Ophthalmologic and Oto-Laryngologic Association was held in Chicago April 7 and 8, 1898. The address of welcome was made by Dr. F. Henrotin, President of the Chicago Medical Society, who, in a felicitous speech, extended to the members the hospitalities of the city of Chicago. Dr. A. Alt, of St. Louis, Mo., responded for the Association. The annual address was then read by President B. E. Friar, of Kansas City, Mo. After the usual routine business had been concluded, a scientific communication was read by Dr. Herman Knapp, of New York City.

The Ophthalmologic and Oto-Laryngologic sections have each held five separate and two joint sessions, many articles of interest being read and discussed. The last joint session was occupied with the exhibition of clinical cases.

The Committee of Arrangements, of which Dr. J. E. Colburn, of Chicago, was chairman, was unremitting in its attention to the guests, and nothing was spared that would contribute to the entertainment of the visitors. Thursday evening the members were invited to the hall of the Chicago Athletic Club, where a special program had been arranged for the entertainment of the members.

The following officers were elected for the ensuing year: President, Dr. J. Elliott Colburn, of Chicago; first vice-president, Dr. W. Scheppegrell, of New Orleans; second vice-president, Dr. Casey A. Wood, of Chicago; third vice-president, Dr. H. Gifford, of Omaha, Neb.; treasurer, Dr. W. L. Dayton, of Lincoln, Neb.; secretary, Dr. F. M. Rumbold, of St. Louis, Mo.

New Orleans was unanimously selected for the next meeting, which will take place just before the Mardi Gras of 1899, thus allowing the members to conclude their scientific session with the gaieties of the carnival season.

---

MOST elaborate preparations are being made in Denver to entertain the next meeting of the American Medical Association which will be held there June 7th to 10th. All indications point to a pleasant and successful meeting, and those who can attend will not fail to enjoy it.

On Saturday, June 4th, the American Academy of Medicine begins its session. Monday, June 6th, the Academy continues its meeting, and on the same day the Medical Editors Association meets, followed by a final session and banquet at night. On Tuesday, June 7th, the general session of the American Medical Association will convene in the Broadway Theater, and in the afternoon the various sections will meet in places provided for them. Tuesday evening the several section banquets will be held.

" On Wednesday there will be another general meeting of the Association and the sections will do hard work. On Wednesday evening a theater party will probably be held at Manhattan Beach.

" On Thursday the regular Association work will close with the election of officers and the sections will finish their work. In the evening a number of receptions will be given at the magnificent homes of some of Denver's wealthy citizens.

"During the three days' sessions the visiting ladies will be cared for in various and divers ways. Rides, excursions, sightseeing and receptions will constitute the program. It will be a merry merry time.

"On Friday, a complimentary excursion will be given around the loop and then our visitors will be sent to Colorado Springs as guests of the local committee of arrangements there. A ride through the Garden of the Gods, a visit to Manitou, to Cascade Canon, to William's Canon, and to Glen Eyre will make a day of the most delightful pleasure. It will be a rare treat. Special arrangements for a trip to the top of Pike's Peak will probably be made.

"After this the members of the Association have the choice of a dozen delightful trips at greatly reduced rates. There will be a great scattering. Salt Lake City is bidding hard for a big portion of the crowd."

Following are the officers of the Association: President, Dr. Geo. M. Sternberg, Surgeon-General United States Army; First Vice-President, Dr. J. M. Mathews, Louisville, Ky.; Second Vice-President, Dr. J. L. Thompson, Indianapolis; Treasurer, Dr. H. P. Newman, Chicago, Ill.; Secretary, Dr. W. B. Atkinson, Philadelphia; Assistant Secretary, Dr. W. A. Jayne, Denver; Chairman of Committee of Arrangements, Dr. J. W. Graham, Denver.

The Address on Medicine will be delivered by Dr. J. H. Musser of Philadelphia. The Address on Surgery will be delivered by Dr. J. B. Murphy of Chicago. Dr. S. C. Busey of Washington, was appointed to deliver the Address on State Medicine, but will not be able to serve. The speaker on this subject has not yet been selected.

